# Associations of psychological wellbeing with COVID-19 hospitalization and mortality in adults aged 50 years or older from 25 European countries and Israel

**DOI:** 10.3389/fpubh.2023.1124915

**Published:** 2023-05-04

**Authors:** Wenjun Wang, Jingjing Wang, Juanjuan Shi, Yaping Li, Xin Zhang, Fengping Wu, Yikai Wang, Jia Li, Miao Hao, Xiongtao Liu, Song Zhai, Yuan Wang, Ning Gao, Yan Tian, Rui Lu, Yee Hui Yeo, Xiaoli Jia, Fanpu Ji, Shuangsuo Dang

**Affiliations:** ^1^Department of Infectious Diseases, Second Affiliated Hospital of Xi'an Jiaotong University, Xi'an, China; ^2^Department of Pediatrics, Second Affiliated Hospital of Xi'an Jiaotong University, Xi'an, China; ^3^Department of Operating Room, Second Affiliated Hospital of Xi'an Jiaotong University, Xi'an, China; ^4^Division of General Internal Medicine, Cedars-Sinai Medical Center, Los Angeles, CA, United States

**Keywords:** COVID-19, psychological wellbeing, CASP-12, SHARE, older people

## Abstract

**Background:**

Lower psychological wellbeing is associated with poor outcomes in a variety of diseases and healthy populations. However, no study has investigated whether psychological wellbeing is associated with the outcomes of COVID-19. This study aimed to determine whether individuals with lower psychological wellbeing are more at risk for poor outcomes of COVID-19.

**Methods:**

Data were from the Survey of Health, Aging, and Retirement in Europe (SHARE) in 2017 and SHARE's two COVID-19 surveys in June–September 2020 and June–August 2021. Psychological wellbeing was measured using the CASP-12 scale in 2017. The associations of the CASP-12 score with COVID-19 hospitalization and mortality were assessed using logistic models adjusted for age, sex, body mass index, smoking, physical activity, household income, education level, and chronic conditions. Sensitivity analyses were performed by imputing missing data or excluding cases whose diagnosis of COVID-19 was solely based on symptoms. A confirmatory analysis was conducted using data from the English Longitudinal Study of Aging (ELSA). Data analysis took place in October 2022.

**Results:**

In total, 3,886 individuals of 50 years of age or older with COVID-19 were included from 25 European countries and Israel, with 580 hospitalized (14.9%) and 100 deaths (2.6%). Compared with individuals in tertile 3 (highest) of the CASP-12 score, the adjusted odds ratios (ORs) of COVID-19 hospitalization were 1.81 (95% CI, 1.41–2.31) for those in tertile 1 (lowest) and 1.37 (95% CI, 1.07–1.75) for those in tertile 2. As for COVID-19 mortality, the adjusted ORs were 2.05 (95% CI, 1.12–3.77) for tertile 1 and 1.78 (95% CI, 0.98–3.23) for tertile 2, compared with tertile 3. The results were relatively robust to missing data or the exclusion of cases solely based on symptoms. This inverse association of the CASP-12 score with COVID-19 hospitalization risk was also observed in ELSA.

**Conclusion:**

This study shows that lower psychological wellbeing is independently associated with increased risks of COVID-19 hospitalization and mortality in European adults aged 50 years or older. Further study is needed to validate these associations in recent and future waves of the COVID-19 pandemic and other populations.

## 1. Introduction

The coronavirus disease 2019 (COVID-19) is a great threat to public health worldwide. As of 16 September 2022, there have been more than 600 million confirmed cases, including 6.5 million deaths (1). Moreover, the pandemic does not show any signs of ending at present. Most severe cases occurred in individuals aged 50 years and older. Compared with younger patients, their hospitalization and mortality rates are 3–5 and 25–340 times higher, respectively (2).

In addition to older age, well-established risk factors for severe COVID-19 include male gender, lower socioeconomic status, poor physical fitness, and underlying diseases such as cardiovascular disease, respiratory disease, cancer, kidney disease, diabetes, and obesity (3–6). As per the WHO's definition of health as “a state of complete physical, mental, and social wellbeing” (7), the above risk factors are related to the physical and social dimensions. However, less attention has been paid to whether factors related to the psychological dimension affect COVID-19 outcomes.

According to human need theory, psychological wellbeing can be measured as the degree that human needs are satisfied (8, 9). Based on this theory, the CASP (control, autonomy, self-realization, and pleasure) scale offers an approach to accessing psychological wellbeing in older people with a meaningful and valid research instrument (8). CASP-12 is the revised 12-item version of the CASP scale. The scale has been translated into 16 languages and used in more than 20 national and international studies (10–15). The objective of this study was to investigate the associations of CASP-12-measured psychological wellbeing with COVID-19 hospitalization and mortality in adults aged 50 years or older in 25 European countries and Israel.

## 2. Materials and methods

### 2.1. Study population

Data were from the Survey of Health, Aging, and Retirement in Europe (SHARE). SHARE is the largest pan-European social science panel study, which every 2 years collected information on health, socioeconomic status, and social and family networks from individuals aged 50 years and older (12). From 2004 until today, SHARE has had eight regular waves, including 140,000 participants from 28 European countries and Israel. In addition to the regular SHARE questionnaire, participants responded to specific questions about COVID-19 infections and changes in life during the pandemic between June and September 2020 (SHARE Corona Survey 1) and between June and August 2021 (SHARE Corona Survey 2) (16, 17). SHARE was reviewed and approved by the Ethics Council of the Max Planck Society (waves 4–8, and SHARE Corona Surveys 1 and 2).

**Figure 1:**
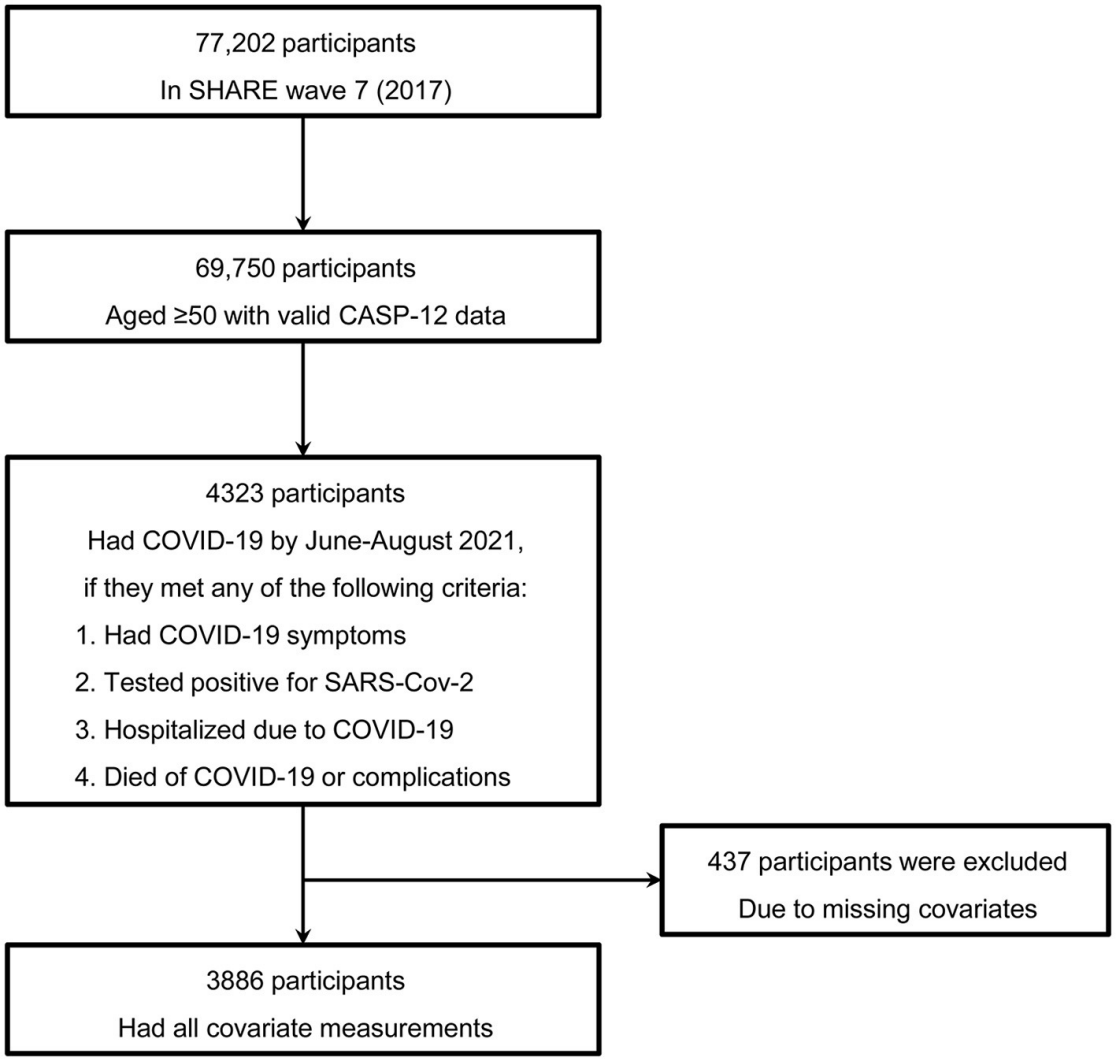
Determination of the study sample. COVID-19, coronavirus disease 2019; SARS-CoV-2, severe acute respiratory syndrome coronavirus 2; SHARE, Survey of Health, Aging, and Retirement in Europe.

In this study, we only included participants in SHARE Wave 7 (conducted in 2017) because this wave had the most recent CASP-12 measurement before the COVID-19 outbreak (18). From the sample of 69,750 participants aged 50 years or older who had valid data for CASP-12 in Wave 7, 4,323 were considered COVID-19 infected according to the subsequent SHARE Corona Surveys 1 and 2. COVID-19 infection was defined if participants had experienced COVID-19 symptoms, had been tested positive for the severe acute respiratory syndrome coronavirus 2 (SARS-CoV-2), had been hospitalized due to COVID-19, or had died of COVID-19 or complications. Only subjects with information available for all covariates were included (missing data were 10.1% for all included covariates), leaving 3,886 individuals for our analyses (Figure 1).

### 2.2. COVID-19 hospitalization and mortality

In SHARE Corona Surveys 1 and 2, participants were asked, “Have you or anyone close to you been hospitalized due to an infection from the Coronavirus?” If participants answered “yes,” they were asked, “Who was hospitalized?” Participants who indicated that they were hospitalized were included in the COVID-19 hospitalization analysis.

SHARE requested interviewers to confirm the decease of a participant by a proxy respondent. In the case of decease, an end-of-life interview was conducted to collect information such as the cause of death. The proxy respondent can be a family/household member, a neighbor, or any other person in the closer social network of the deceased participant. Participants who died of COVID-19 or related complications were included in the analysis of COVID-19 mortality. These deceased participants also constituted the sample of COVID-19 hospitalization because they usually died in hospitals or other health facilities.

### 2.3. Psychological wellbeing

Psychological wellbeing was measured using the CASP-12 scale (8, 19). The CASP-12 is a 12-item scale composed of four domains, such as control, autonomy, self-realization, and pleasure. Each domain has three items, which are presented as questions or statements to survey participants. Each item is assessed on a 4-point Likert scale (“often,” “sometimes,” “rarely,” and “never”) (Supplementary Table S1). The resulting score is the sum of these 12 items and ranges from a minimum of 12 to a maximum of 48 (19). A high score indicates a high level of psychological wellbeing. The literature does not indicate a threshold that categorizes psychological wellbeing as “low” or “high.”

### 2.4. Covariates

Potential confounding factors were included in the analyses as follows: age (when interviewed in SHARE wave 7 in 2017), sex, body mass index, smoking status, physical activity, household income, education, and underlying health conditions. Body mass index was calculated as weight/height^2^ and was classified into categories of underweight (< 18.5), normal weight (18.5 to < 25), overweight (25 to < 30), and obese (≥30), according to the WHO's criteria. Height and weight were self-reported in SHARE. Participants were asked how many days per week they engaged in moderate (e.g., gardening, cleaning the car, or doing a walk) and vigorous-intensity physical activities (e.g., sports, heavy housework, or a job that involves physical labor). If participants answered “Hardly ever, or never,” they were considered physically inactive. Participants were asked, “Have you ever smoked cigarettes, cigars, cigarillos, or a pipe daily for a period of at least one year?” If participants answered “Yes,” they were classified as ever smoked daily. Education level was coded according to the International Standard Classification of Education-97 (ISCED-97) criteria and classified as low level (no education or ISCED-97 codes 1 and 2), middle level (ISCED-97 codes 3 and 4), and high level (ISCED-97 codes 5 and 6). Household income was categorized into country-specific quartiles. The following health conditions were asked whether participants ever diagnosed or had at the time of the interview: respiratory diseases (such as chronic bronchitis or emphysema), cardiovascular diseases (heart attack including myocardial infarction or coronary thrombosis or any other heart problem including congestive heart failure and stroke or cerebral vascular disease), diabetes or high blood sugar, cancer or malignant tumor (including leukemia or lymphoma but excluding minor skin cancer), chronic kidney disease, and rheumatoid arthritis.

### 2.5. Statistical analyses

A total of four logistic regression models were fitted to test the associations of the CASP-12 score with COVID-19 hospitalization and mortality. Model 0 was unadjusted. Model 1 adjusted for age (50–60, 60–70, >70 years) and sex. Additionally, Model 2 adjusted for body mass index, smoking, physical activity, household income, and education level. Finally, Model 3 further adjusted for underlying health conditions including respiratory disease, cardiovascular disease, diabetes, cancer, chronic kidney disease, and rheumatoid arthritis. Interaction terms were fitted to Model 3 to assess whether age (50–60, 60–70, >70 years) and sex modified associations with COVID-19 hospitalization and mortality.

First, odds ratios (ORs) for age-specific and sex-specific tertiles of the CASP-12 score were calculated, with participants in the highest tertile for the CASP-12 score used as the reference group. Linear associations between continuous independent variables and COVID-19 outcomes were checked using the Box-Tidwell test. As no evidence of deviation from linearity was found, the CASP-12 score was also treated as a continuous variable in the above models, and ORs were calculated per score decrement in the score.

A total of two sensitivity analyses were conducted. In the first sensitivity analysis, participants whose diagnosis of COVID-19 infection was solely based on symptoms were excluded. Thus, the included participants were those who tested positive for SARS-Cov-2, or hospitalized due to COVID-19, or died of COVID-19 or related complications. In the second sensitivity analysis, missing values of covariates were imputed (Supplementary Method S1).

A confirmatory analysis was conducted to validate the association between CASP-12 and COVID-19 hospitalization using data from the English Longitudinal Study of Aging (ELSA) (13). ELSA is a panel survey of people aged 50 years and older living in England. It has been carried out every 2 years since 2002 to collect data on health, economic, and social circumstances and now has nine regular waves. Wave 9, conducted in 2018–2019, is the most recent regular wave before the outbreak of COVID-19. During 2020, two additional waves (COVID-19 Wave 1, June–July 2020; COVID-19 Wave 2, November–December 2020) were conducted to collect information on the impact of the COVID-19 crisis on health, social care, financial circumstances, and social activity. Of the 6,965 participants aged 50 years and older with valid data for CASP-12 in ELSA Wave 9, 285 had COVID-19 infection and were included in the confirmatory analysis (Supplementary Figure S). The association between CASP-12 and COVID-19 mortality was not assessed because end-of-life data are currently not released. Ethical approval for ELSA Wave 9 was granted by the South Central Berkshire Research Ethics Committee through an application to the National Research Ethics Service. ELSA COVID-19 waves 1 and 2 were reviewed and approved by the University College London Research Ethics Committee. Detailed methods of confirmatory analysis are presented in Supplementary Method S2. ORs were accompanied by corresponding 95% confidence intervals (95% CIs). All analyses were performed with StataSE 15 (Stata Corporation, College Station, TX, United States).

## 3. Results

### 3.1. Characteristics of the study population

Of the 3,886 participants with COVID-19 infection included in the study, 1,607 (41.4%) participants were men and 2,277 (58.6%) were women. The mean (range) age was 65.5 (50.1–96.3) years. The 3,886 participants were from 25 European countries and Israel. By June–August 2021, 580 (14.9%) participants were hospitalized and 100 (2.6%) died due to COVID-19.

**Figure 2:**
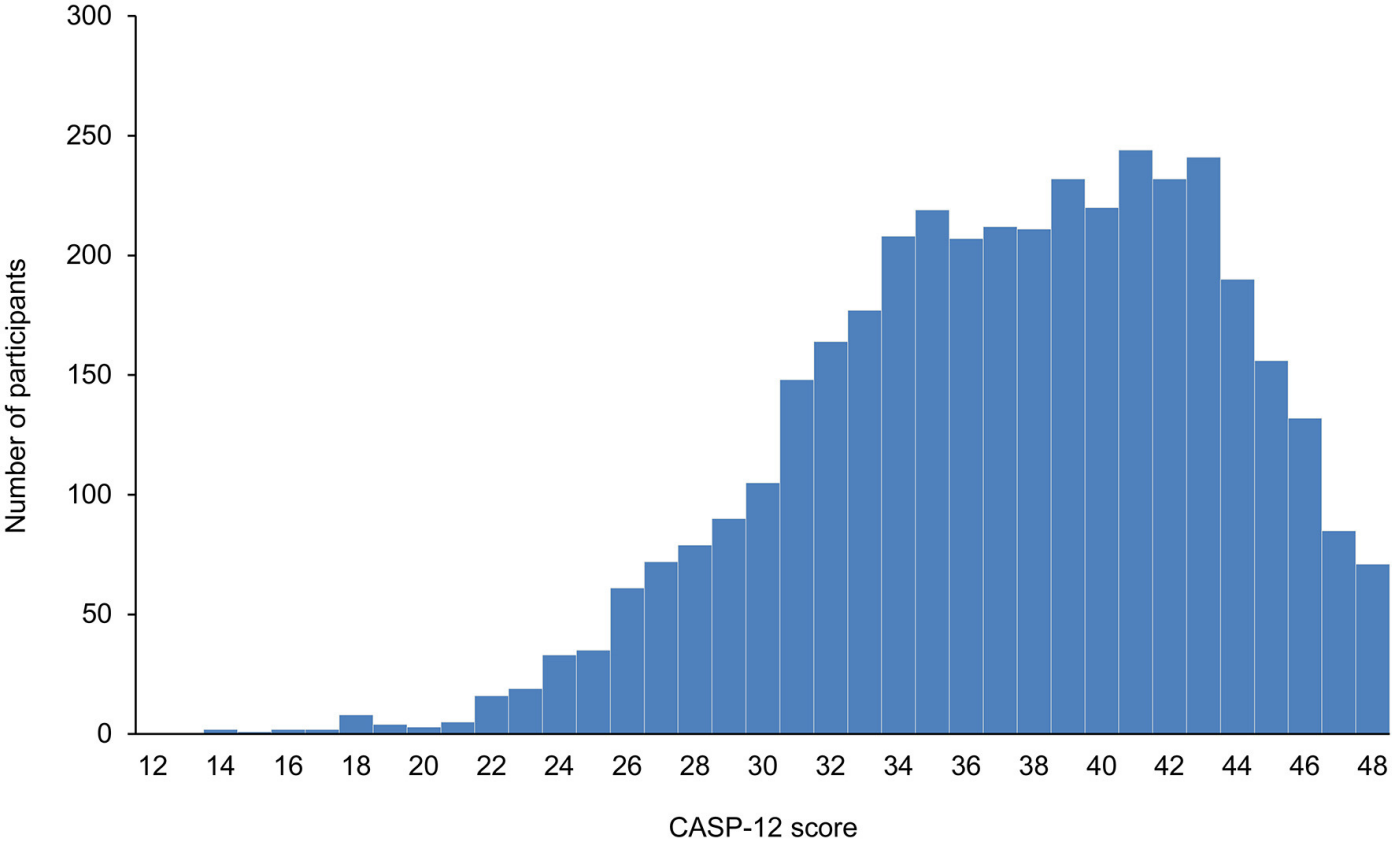
Distribution of the CASP-12 score of the 3,886 participants.

The distribution of the CASP-12 score of the 3,886 participants is shown in Figure 2. The score ranged from 14 to 48, with a median of 38 (interquartile range [IQR]: 33–42). Individuals who were hospitalized or died due to COVID-19 had lower CASP-12 scores before the COVID-19 outbreak compared with those not (median score: 36 (IQR, 31–40) vs. 38 (IQR, 34–42) for hospitalization; 35 (IQR, 30–39) vs. 38 (IQR, 33–42) for mortality; *P* < 0.0001 for each, Mann–Whitney *U*-test).

Table 1 summarizes the main characteristics of the participants by age- and sex-specific tertiles of the CASP-12 score. In brief, people in the lowest tertile (tertile 1) for the CASP-12 score had lower levels of education, household income, and physical activity and had a higher prevalence of obesity and comorbidities, including cardiovascular disease, respiratory disease, diabetes, cancer, chronic kidney disease, and rheumatoid arthritis, compared with the highest CASP-12 score group (tertile 3) (*P* < 0.01 for each, chi-square test).

**Table 1:** Characteristics of the cohort by age- and sex-specific tertiles of CASP-12 score.

**Characteristics**	**All (*n* = 3,886)**	**Tertile of CASP-12 score**		
		**Tertile 1 (lowest) (*****n*** = **1,347)**	**Tertile 2 (*****n*** = **1,252)**	**Tertile 3 (highest) (*****n*** = **1,287)**
Gender, male	41.4% (1,609)	42.0% (566)	41.1% (515)	41.0% (528)
Age, mean (SD), years	65.5 (8.6)	65.8 (9.0)	65.6 (8.5)	65.3 (8.3)
**Body mass index categories**				
Underweight (< 18.5)	0.7% (27)	0.7% (9)	0.5% (6)	0.9% (12)
Normal weight (18.5 to < 25)	29.4% (1,141)	24.5% (330)	29.6% (370)	34.3% (441)
Overweight (25 to < 30)	40.9% (1,590)	41.6% (560)	41.5% (520)	39.6% (510)
Obese (≥30)	29.0% (1,128)	33.3% (448)	28.4% (356)	25.2% (324)
**Education level**				
Low	30.2% (1,174)	38.2% (514)	29.2% (365)	22.9% (295)
Middle	45.2% (1,756)	44.3% (597)	46.1% (577)	45.2% (582)
High	24.6% (956)	17.5% (236)	24.8% (310)	31.9% (410)
**Household income**				
Quartile 1 (lowest)	19.8% (768)	27.0% (363)	18.1% (226)	13.9% (179)
Quartile 2	24.0% (934)	27.7% (373)	22.4% (280)	21.8% (281)
Quartile 3	26.4% (1,025)	23.5% (317)	29.6% (370)	26.3% (338)
Quartile 4 (highest)	29.8% (1,159)	21.8% (294)	30.0% (376)	38.0% (489)
Physical inactivity	9.3% (360)	15.4% (207)	7.8% (97)	4.4% (56)
Ever smoked daily	42.2% (1,638)	42.6% (574)	41.4% (518)	42.4% (546)
Cardiovascular disease	13.6% (530)	19.6% (264)	13.2% (165)	7.9% (101)
Respiratory disease	5.2% (203)	6.5% (88)	5.7% (71)	3.4% (44)
Diabetes	12.3% (479)	15.7% (211)	11.0% (138)	10.1% (130)
Cancer	4.4% (170)	5.8% (78)	4.7% (59)	2.6% (33)
Chronic kidney disease	2.2% (86)	3.0% (41)	2.7% (34)	0.9% (11)
Rheumatoid arthritis	10.6% (412)	15.1% (204)	10.0% (125)	6.5% (83)

Values are percentages (numbers) unless stated otherwise.

### 3.2. Associations of established risk factors with COVID-19 hospitalization and mortality

Older age, male gender, physical inactivity, lower levels of education, diabetes, and being overweight or obese were associated with an increased risk of COVID-19 hospitalization. Older age, male gender, physical inactivity, and diabetes were associated with an increased risk of COVID-19 mortality. The other associations were not statistically significant (Supplementary Table S2).

### 3.3. Associations of CASP-12 with COVID-19 hospitalization and mortality

As shown in Table 2, individuals in tertile 1 (the lowest) and tertile 2 (the medium) of the CASP-12 score had higher risks of COVID-19 hospitalization and mortality compared with those in tertile 3 (the highest) in Model 0. After adjustment for age and sex, the risks were similar in Model 1; after further adjustment, the magnitude of the risks was slightly attenuated in Model 2 and Model 3. In the fully adjusted model (Model 3), compared with individuals in tertile 3, the ORs of COVID-19 hospitalization were 1.81 (95% CI, 1.41–2.31) for those in tertile 1 and 1.37 (95% CI, 1.07–1.75) for those in tertile 2. As for COVID-19 mortality, the fully adjusted ORs in Model 3 were 2.05 (95% CI, 1.12–3.77) for tertile 1 vs. tertile 3 and 1.78 (95% CI, 0.98–3.23) for tertile 2 vs. tertile 3.

**Table 2:** Associations of tertile CASP-12 score with COVID-19 hospitalization and mortality.

**Model**	**COVID-19 hospitalization, OR (95% CI)**		**COVID-19 mortality, OR (95% CI)**	
	**Tertile 1 vs. Tertile 3**	**Tertile 2 vs. Tertile 3**	**Tertile 1 vs. Tertile 3**	**Tertile 2 vs. Tertile 3**
0	1.92 (1.54–2.40)	1.41 (1.12–1.79)	2.49 (1.44–4.32)	2.09 (1.18–3.70)
1	1.98 (1.58–2.49)	1.44 (1.13–1.84)	2.55 (1.46–4.46)	2.12 (1.19–3.79)
2	1.77 (1.39–2.25)	1.38 (1.08–1.77)	2.00 (1.10–3.62)	1.92 (1.07–3.45)
3	1.81 (1.41–2.31)	1.37 (1.07–1.75)	2.05 (1.12–3.77)	1.78 (0.98–3.23)

Model adjustment.

Model 0: unadjusted.

Model 1: age and sex.

Model 2: Model 1 + body mass index, smoking, physical activity, household income, and education level.

Model 3: Model 2 + respiratory disease, cardiovascular disease, diabetes, cancer, chronic kidney disease, and rheumatoid arthritis.

CI, confidence interval; OR, odds ratio.

When the CASP-12 score was treated as a continuous variable, similar results for COVID-19 hospitalization and mortality were found (Supplementary Table S3). The fully adjusted ORs per score decrement in CASP-12 were 1.03 (95% CI, 1.02–1.05) and 1.04 (95% CI, 1.01–1.08) for COVID-19 hospitalization and mortality, respectively. Age and sex had no significant interaction effects on the associations of CASP-12 score with COVID-19 hospitalization and mortality (P for interaction >0.05).

### 3.4. Sensitivity analyses

In the first sensitivity analysis, 1,187 participants whose diagnosis of COVID-19 infection was solely based on symptoms were excluded, leaving 2,699 participants with 580 hospitalized and 100 deaths. The magnitude of the risks for COVID-19 hospitalization and mortality regarding the CASP-12 score hardly changed, either being treated as a tertile cutoff (Supplementary Table S4) or as a continuous variable (Supplementary Table S5). In the second sensitivity analysis, after imputing for missing data of covariates, there were 4,323 participants with COVID-19 infection, including 646 hospitalized and 111 deaths. The magnitude of the risks was only slightly attenuated (Supplementary Table S6).

### 3.5. Confirmatory analyses in the ELSA Cohort

Of the 285 participants with COVID-19 infection in the ELSA cohort, 108 (37.9%) were men and 177 (62.1%) were women. The median age was 65 (IQR, 56–72) years. By November–December 2020, 37 (13.0%) participants were hospitalized due to COVID-19. The score of CASP-12 ranged from 17 to 48, with a median of 38 (IQR, 33–42). Individuals who were hospitalized due to COVID-19 tended to have lower CASP-12 scores before the COVID-19 outbreak compared with those not (median score: 35 (IQR, 29–41) vs. 38 (IQR, 34–42), *P* = 0.067, Mann–Whitney *U*-test). The unadjusted OR per score decrement in CASP-12 was 1.06 (95% CI, 1.00–1.11) for COVID-19 hospitalization. The association (OR, 1.06; 95% CI, 1.01–1.13) did not change after adjustment for age and sex. As only 37 subjects were hospitalized, adjustment for more covariates was not performed.

## 4. Discussion

The main finding of this study is that a lower CASP-12 score was associated with hospitalization and mortality in COVID-19-infected individuals aged 50 years and older from 25 European countries and Israel. The associations observed were relatively robust after adjustment for established risk factors for severe COVID-19, including older age, male gender, lower socioeconomic status, poor physical fitness, and underlying diseases such as cardiovascular disease, respiratory disease, cancer, kidney disease, diabetes, and obesity. The association of CASP-12 with COVID-19 hospitalization was confirmed in the English population.

To the best of our knowledge, this is the first study to investigate how psychological wellbeing affects clinical outcomes of COVID-19 infection. Previous studies mainly focused on the impact of the COVID-19 pandemic on psychological wellbeing, and most studies showed a negative effect on people's psychological wellbeing for those who were infected and for those who were not infected (20). Prospective studies of populations with other diseases showed that positive psychological wellbeing is associated with favorable physical health outcomes (21–26). These diseases include cancer, cardiovascular disease, renal failure, human immunodeficiency virus infection, and patients undergoing major surgery. Involved measures of psychological wellbeing include emotional wellbeing, positive mood, joy, happiness, vigor, energy, life satisfaction, hopefulness, optimism, and a sense of humor. Data from ELSA, a general aged population in England, showed that compared with the lowest quartile, the highest quartile of CASP-19 (a 19-item CASP scale) score was associated with a 30% (95% CI 16.7–41.7%) reduction in mortality risk after adjusting for age, sex, education and wealth, health status, measures of depression, and health behaviors such as smoking, physical activity, and alcohol consumption (27). As for COVID-19, the current study revealed a similar trend for disease outcomes concerning the CASP-12 scale. Taken together, increasing psychological wellbeing is not only a goal in itself for a human being, but may also be a promising non-biological approach to improving outcomes in healthy and diseased populations, including those with COVID-19.

The current population-based study implies the potential value of CASP-12 in predicting the prognosis of COVID-19. Moreover, the CASP-12 scale was developed for older people (8). Thus, this tool may be more advantageous in COVID-19 than other tools assessing psychological wellbeing, since severe disease and mortality mainly occur in older people. In addition, the 12-item questionnaire can be easily performed, and the respondent burden is low. The CASP-12 has demonstrated good validity and reliability and now has been widely adopted, particularly in large surveys of aging populations (10–15). Future studies should assess its prognostic value in clinical settings.

Behavioral pathways are thought to partly mediate the association between psychological wellbeing and clinical outcomes. For example, negative psychological wellbeing, which is characterized by low levels of positive emotions, high levels of negative emotions, and a lack of life satisfaction, is related to smoking, drinking, low physical activity levels, poor sleep quality, and eating fewer fruits and vegetables (27, 28). The latter are well-established predictors for mortality and morbidity. In the current study, individuals with lower CASP-12 scores had lower levels of physical activity. After adjusting for physical activity, the ORs of COVID-19 hospitalization and mortality regarding CASP-12 were slightly attenuated. Future studies are suggested to include other behavioral factors.

Some biological mechanisms may be involved in the association between psychological wellbeing and COVID-19 outcomes. Negative wellbeing is related to increased levels of cortisol (29, 30), which is a marker of the severity of many diseases, such as pneumonia (31). Evidence supports high cortisol levels as an independent predictor of COVID-19 severity and mortality (32). Negative wellbeing is also associated with stress-induced elevations of inflammation, such as C-reactive protein, interleukin-6, fibrinogen, and white blood cell (29, 30, 33), which are biomarkers of critical COVID-19 and associated with mortality (34–36).

The first strength of the study is the longitudinal design of SHARE and its representative sample of Europeans aged 50 years or older. Second, the OR values suggested the associations of the CASP-12 score with COVID-19 hospitalization and mortality are relatively strong. A dose-dependent effect was suggested from logistic regressions when the CASP-12 score was treated as a continuous variable. A higher OR value for tertile 1 vs. tertile 3 than that for tertile 2 vs. tertile 3 also showed the trend. Third, the associations are relatively robust to missing data or the exclusion of cases solely based on symptoms. The association of CASP-12 with COVID-19 hospitalization was externally confirmed in the ELSA cohort.

The study has some limitations. First, information on COVID-19 infection, hospitalization, mortality, and covariates was collected with questionnaires. They are not as accurate as data from medical records, mortality registers, and direct measurements. Psychological wellbeing was measured in 2017 which was a significant period before the COVID-19 outbreak. Second, although a wide range of demographic, lifestyle, socioeconomic, and clinical factors were adjusted, other unmeasured factors that could potentially confound the observed associations cannot be ruled out. Third, in the validation cohort ELSA, the sample size was small and not allowed to adjust for more covariates, and due to the lack of mortality data, the association of the CASP-12 score with COVID-19 mortality was not validated. Fourth, information was lacking on COVID-19 vaccination and strains of SARS-Cov-2, which are associated with the disease severity. People's psychological status, as well as their socioeconomic status, living conditions, behavior, and lifestyle, have been profoundly changed by the pandemic. The shortage of health resources at the early stages of the pandemic has been alleviated to some extent now (37, 38). Taken together, whether the findings of the current study will change in recent and future waves of the COVID-19 pandemic needs further evaluation.

In conclusion, this study shows that lower psychological wellbeing measured on the CASP-12 scale is independently associated with increased risks of COVID-19 hospitalization and mortality in European adults aged 50 years or older. Further study is needed to validate these associations in recent and future waves of the COVID-19 pandemic and in other populations. If this is the case, promoting psychological wellbeing may be a potential approach to improving the disease outcomes in patients with older age, the most vulnerable subgroup of COVID-19.

## Data availability statement

The raw data supporting the conclusions of this article will be made available by the authors, without undue reservation.

## Ethics statement

The studies involving human participants were reviewed and approved by the Ethics Council of the Max Planck Society. The patients/participants provided their written informed consent to participate in this study.

## Author contributions

WW, XJ, FJ, and SD conceived and designed the study. WW and JW acquired the data and conducted the statistical analyses. JS and YL verified the data. WW, JW, JS, and YL drafted the manuscript. All authors interpreted the data, reviewed and contributed revisions to the final version of the manuscript, and approved the final version of the manuscript.
