# Supplementary material for: Associations of psychological wellbeing with COVID-19 hospitalization and mortality in adults aged 50 years or older from 25 European countries and Israel
Source: Front Public Health. 2023 May 4;11:1124915. doi: 10.3389/fpubh.2023.1124915 (PMC10194836; doi:10.3389/fpubh.2023.1124915)
Supplement: Supplementary file 1 [file Data_Sheet_1.docx]

Supplementary Material

Associations of Psychological Wellbeing with COVID-19 Hospitalization and Mortality in Adults 50 Years of Age or Older from 25 European Countries and Israel

**Wenjun Wang^1^, Jingjing Wang^2^, Juanjuan Shi^1^, Yaping Li^1^, Xin Zhang^1^, Fengping Wu^1^, Yikai Wang^1^, Jia Li^1^, Miao Hao^1^, Xiongtao Liu^3^, Song Zhai^1^, Yuan Wang^1^, Ning Gao^1^, Yan Tian^1^, Rui Lu^1^, Yee Hui Yeo^4^, Xiaoli Jia^1*^, Fanpu Ji^1*^, Shuangsuo Dang^1*^**

*** Correspondence:** Xiaoli Jia: drjxl@163.com; Fanpu Ji: jifanpu1979@163.com; Shuangsuo Dang: dangshuangsuo123@xjtu.edu.cn

**Table S1.** Item wording for CASP-12

**Method S1.** Imputation methods for missing values used in the sensitivity analyses

**Figure S.** Determination of the study sample for confirmatory analysis

**Method S2.** Methods for examing the association of CASP-12 with COVID-19 hospitalization in ELSA cohort

**Table S2.** Associations of established risk factors with COVID-19 hospitalization and mortality

**Table S3.** Associations of CASP-12 with COVID-19 hospitalization and mortality (CASP-12 as a continuous variable)

**Table S4.** Sensitivity analyses after excluding participants whose diagnosis of COVID-19 infection was solely based on symptoms (tertiled CASP-12 score)

**Table S5.** Sensitivity analyses after excluding participants whose diagnosis of COVID-19 was solely based on symptoms (CASP-12 as a continuous variable)

**Table S6.** Sensitivity analyses for the associations of CASP-12 with COVID-19 hospitalization and mortality after imputing for missing data of covariates

**Table S1. Item wording for CASP-12**

| **Domain** | **Items** |
| --- | --- |
| Control | 1. How often do you think your age prevents you from doing the things you would like to do? |
|  | 2. How often do you feel that what happens to you is out of your control? |
|  | 3. How often do you feel left out of things? |
|  |  |
| Autonomy | 4. How often do you think that you can do the things that you want to do? |
|  | 5. How often do you think that family responsibilities prevent you from doing what you want to do? |
|  | 6. How often do you think that shortage of money stops you from doing the things you want to do? |
|  |  |
| Pleasure | 7. How often do you look forward to each day? |
|  | 8. How often do you feel that your life has meaning? |
|  | 9. How often, on balance, do you look back on your life with a sense of happiness? |
|  |  |
| Self-Realization | 10. How often do you feel full of energy these days? |
|  | 11. How often do you feel that life is full of opportunities? |
|  | 12. How often do you feel that the future looks good for you? |

Response options are “often”, “sometimes”, “rarely”, and “never”. Negatively worded items (items 1, 2, 3, 5, and 6) are scored 1-4; positively worded items (items 4, 7, 8, 9, 10, 11, and 12) are scored 4-1.

**Method S1. Imputation methods for missing values used in the sensitivity analyses**

In total, 437 out of 4323 participants (10.1%) from the SHARE cohort were excluded from the main analysis due to missing values of covariates. In the sensitivity analysis, the missing values were imputed as follows:

First, missing values for body mass index and education level were imputed using their mean values. Then, a multiple imputation method using chained equations was used to impute missing values for physical activity, smoking, cardiovascular disease, respiratory disease, diabetes, cancer, chronic kidney disease, and rheumatoid arthritis. In the chained equations, the logistic method was used as the univariate method, and age (50-60, 60-70, >70) and gender were input as independent variables.

**Figure S. Determination of the study sample for confirmatory analysis.**


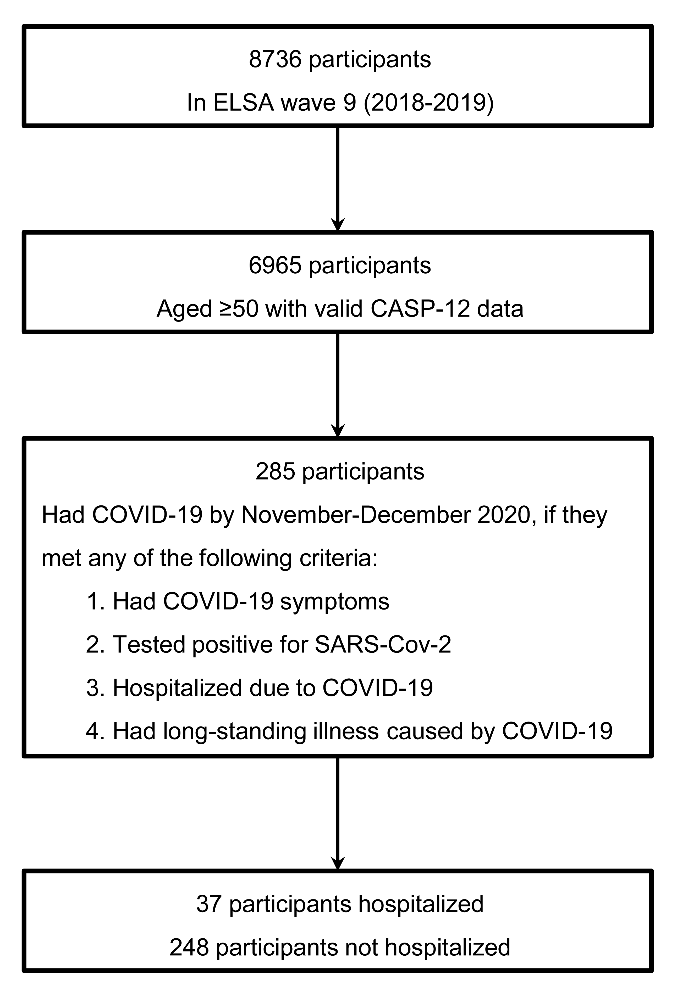


COVID-19, coronavirus disease 2019; SARS-CoV-2, severe acute respiratory syndrome coronavirus 2; ELSA, the English Longitudinal Study of Ageing.

**Method S2. Methods for examing the association of CASP-12 with COVID-19 hospitalization in ELSA cohort**

ELSA is a panel survey of people aged 50 and over living in England. It has been carried out every two years since 2002 to collect data on health, economic, and social circumstances and now has nine regular waves. Wave 9, conducted in 2018-2019, is the most recent regular wave before the outbreak of COVID-19. During 2020, two additional waves (COVID-19 Wave 1, June-July 2020; COVID-19 Wave 2, November-December 2020) were conducted to collect information on the impact of the COVID-19 crisis on health, social care, financial circumstances, and social activity. Of the 6965 participants aged 50 and over with valid data for CASP-12 in ELSA Wave 9, 285 had COVID-19 infection and were included in the confirmatory analysis. The association between CASP-12 and COVID-19 mortality was not assessed because end-of-life data are currently not released. Ethical approval for ELSA Wave 9 was granted by the South Central Berkshire Research Ethics Committee through an application to the National Research Ethics Service. ELSA COVID-19 waves 1 and 2 were reviewed and approved by the University College London Research Ethics Committee. Detailed methods of confirmatory analysis are described in eMethod 2 in the Supplement.

In COVID-19 Wave 1, COVID-19 infection was considered if participants answered “I have or had symptoms of coronavirus.” to the question “Why were you self-isolating? (CvSelfWhy1)”, or “It was positive” to the question “What was the result of your coronavirus test? (CvTestB)”, or “Yes” to the question “Have you had to stay in hospital for treatment due to coronavirus? (CvHosp)”. In COVID-19 Wave 2, participants were considered COVID-19 infection if they answered “Positive” to the question “What was the result of your coronavirus test? (CvTestB)”, or “Yes” to the question “Since we last interviewed you have you had to stay in hospital for treatment due to coronavirus? (CvHosp)”, or “Yes/No” to the question “Have you been told by a doctor that you have any long-standing illness or disability caused by coronavirus? (CvLongCovid)”.

COVID-19 hospitalization was considered if participants answered “Yes” to the question “Have you had to stay in hospital for treatment due to coronavirus? (CvHosp)” in COVID-19 wave 1, or “Yes” to the question “Since we last interviewed you have you had to stay in hospital for treatment due to coronavirus? (CvHosp)”.

ELSA Wave 9 had the score for each item of CASP-19, the 19-item version of CASP. CASP-12 is a shortened 12-item version of CASP-19. The CASP-12 score was calculated as in SHARE (Supplementary data Table S1).

Logistic regression models were fitted to test the associations of the CASP-12 score with COVID-19 hospitalization. First, an unadjusted model was used. Then, age and sex were adjusted. As only 37 subjects were hospitalized, other covariates were not adjusted further.

**Table S2. Associations of established risk factors with COVID-19 hospitalization and mortality**

| **Factor** | **COVID-19 hospitalization**  **OR (95% CI)** | **COVID-19 mortality**  **OR (95% CI)** |
| --- | --- | --- |
| Age (50-60, 60-70, >70) | 2.00 (1.75-2.30) | 3.98 (2.68-5.90) |
| Overweight or obese (BMI ≥25) | 1.41 (1.14-1.76) | 1.21 (0.73-1.99) |
| Female | 0.62 (0.51-0.75) | 0.30 (0.19-0.49) |
| Education level (from low to high) | 0.81 (0.71-0.93) | 0.80 (0.59-1.09) |
| Physical active | 0.56 (0.43-0.73) | 0.40 (0.24-0.65) |
| Household income (from quartile 1 to 4) | 1.00 (0.91-1.10) | 0.98 (0.79-1.21) |
| Ever smoked daily | 1.04 (0.86-1.26) | 0.90 (0.59-1.40) |
| Cardiovascular disease | 1.21 (0.95-1.54) | 1.10 (0.68-1.80) |
| Respiratory disease | 1.09 (0.75-1.59) | 1.28 (0.61-2.68) |
| Diabetes | 1.31 (1.02-1.68) | 1.84 (1.15-2.95) |
| Cancer | 0.89 (0.57-1.37) | 1.52 (0.71-3.22) |
| Chronic kidney disease | 1.25 (0.72-2.16) | 0.82 (0.24-2.83) |
| Rheumatoid arthritis | 0.77 (0.56-1.05) | 0.91 (0.47-1.78) |

BMI, body mass index; CI, confidence interval; OR, odds ratio.

**Table S3. Associations of CASP-12 with COVID-19 hospitalization and mortality (CASP-12 as a continuous variable)**

| **Model** | **COVID-19 hospitalization**  **OR (95% CI)** | **COVID-19 mortality**  **OR (95% CI)** |
| --- | --- | --- |
| 0 | 1.05 (1.04-1.07) | 1.08 (1.04-1.11) |
| 1 | 1.04 (1.03-1.06) | 1.07 (1.03-1.10) |
| 2 | 1.03 (1.02-1.05) | 1.05 (1.01-1.08) |
| 3 | 1.04 (1.02-1.05) | 1.04 (1.01-1.08) |

Model 0: unadjusted.

Model 1: age and sex.

Model 2: Model 1 + body mass index, smoking, physical activity, household income, and education level.

Model 3: Model 2 + respiratory disease, cardiovascular disease, diabetes, cancer, chronic kidney disease, and rheumatoid.

CI, confidence interval; OR, odds ratio.

**Table S4. Sensitivity analyses after excluding participants whose diagnosis of COVID-19 infection was solely based on symptoms (tertiled CASP-12 score)**

| **Model** | **COVID-19 hospitalization, OR (95% CI)** | |  | **COVID-19 mortality, OR (95% CI)** | |
| --- | --- | --- | --- | --- | --- |
|  | **Tertile 1 vs. Tertile 3** | **Tertile 2 vs. Tertile 3** |  | **Tertile 1 vs. Tertile 3** | **Tertile 2 vs. Tertile 3** |
| 0 | 1.94 (1.54-2.44) | 1.46 (1.14-1.86) |  | 2.43 (1.40-4.22) | 2.11 (1.19-3.75) |
| 1 | 2.01 (1.59-2.56) | 1.46 (1.13-1.88) |  | 2.47 (1.41-4.35) | 2.05 (1.14-3.69) |
| 2 | 1.85 (1.43-2.38) | 1.45 (1.12-1.88) |  | 2.01 (1.10-3.67) | 1.93 (1.06-3.50) |
| 3 | 1.88 (1.45-2.43) | 1.40 (1.08-1.82) |  | 1.99 (1.07-3.68) | 1.74 (0.95-3.21) |

Model adjustment.

Model 0: unadjusted.

Model 1: age and sex.

Model 2: Model 1 + body mass index, smoking, physical activity, household income, and education level.

Model 3: Model 2 + respiratory disease, cardiovascular disease, diabetes, cancer, chronic kidney disease, and rheumatoid arthritis.

CI, confidence interval; OR, odds ratio.

**Table S5. Sensitivity analyses after excluding participants whose diagnosis of COVID-19 was solely based on symptoms (CASP-12 as a continuous variable)**

| **Model** | **COVID-19 hospitalization**  **OR (95% CI)** | **COVID-19 mortality**  **OR (95% CI)** |
| --- | --- | --- |
| 0 | 1.05 (1.04-1.07) | 1.08 (1.05-1.11) |
| 1 | 1.05 (1.03-1.06) | 1.07 (1.03-1.10) |
| 2 | 1.04 (1.02-1.06) | 1.05 (1.01-1.09) |
| 3 | 1.04 (1.02-1.05) | 1.04 (1.01-1.08) |

Model adjustment.

Model 0: unadjusted.

Model 1: age and sex.

Model 2: Model 1 + body mass index, smoking, physical activity, household income, and education level.

Model 3: Model 2 + respiratory disease, cardiovascular disease, diabetes, cancer, chronic kidney disease, and rheumatoid

CI, confidence interval; OR, odds ratio.

**Table S6. Sensitivity analyses for the associations of CASP-12 with COVID-19 hospitalization and mortality after imputing for missing data of covariates**

| **CASP-12** | **COVID-19 hospitalization**  **OR (95% CI)** | **COVID-19 mortality**  **OR (95% CI)** |
| --- | --- | --- |
| Tertile 1 (lowest) vs. Tertile 3 (highest) | 1.70 (1.35-2.15) | 1.71 (0.96-3.05) |
| Tertile 2 (medium) vs. Tertile 3 (highest) | 1.35 (1.07-1.70) | 1.65 (0.95-2.87) |
| CASP-12 as a continuous variable | 1.03 (1.02-1.05) | 1.04 (1.01-1.08) |

Adjusted for age, sex, body mass index, smoking, physical activity, household income, education level, respiratory disease, cardiovascular disease, diabetes, cancer, chronic kidney disease, and rheumatoid arthritis.

CI, confidence interval; OR, odds ratio.
